# Transcriptional Expression in Human Periodontal Ligament Cells Subjected to Orthodontic Force: An RNA-Sequencing Study

**DOI:** 10.3390/jcm9020358

**Published:** 2020-01-28

**Authors:** Kyunam Kim, Hee Eun Kang, Jong In Yook, Hyung-Seog Yu, Euiseong Kim, Jung-Yul Cha, Yoon Jeong Choi

**Affiliations:** 1 Department of Orthodontics, The Institute of Craniofacial Deformity, Yonsei University College of Dentistry, Seoul 03722, Korea; novice0801@naver.com (K.K.); yumichael@yuhs.ac (H.-S.Y.); jungcha@yuhs.ac (J.-Y.C.); 2 Vatech Co., Ltd. Hwaseong-si, Gyeonggi-do 18449, Korea; wing870817@gmail.com; 3 Department of Oral Pathology, Oral Cancer Research Institute, Yonsei University College of Dentistry, Seoul 03722, Korea; jiyook@yuhs.ac; 4 Department of Conservative Dentistry, Oral Science Research Center, Yonsei University College of Dentistry, Seoul 03722, Korea; andyendo@yuhs.ac

**Keywords:** RNA-sequencing, orthodontic force, periodontal ligament, mechanical stimulus, prospective study

## Abstract

This study was performed to investigate the changes in gene expression in periodontal ligament (PDL) cells following mechanical stimulus through RNA sequencing. In this study, premolars extracted for orthodontic treatment were used. To stimulate the PDL cells, an orthodontic force of 100*× g* was applied to the premolar (experimental group; *n* = 11), whereas the tooth on the other side was left untreated (control group; *n* = 11). After the PDL cells were isolated from the extracted teeth, gene set enrichment analysis (GSEA), differentially expressed gene (DEG) analysis, and real-time PCR were performed to compare the two groups. GSEA demonstrated that gene sets related to the cell cycle pathway were upregulated in PDL. Thirteen upregulated and twenty downregulated genes were found through DEG analysis. Real-time PCR results confirmed that five upregulated genes (*CC2D1B*, *CPNE3*, *OPHN1*, *TANGO2*, and *UAP-1*) and six downregulated genes (*MYOM2*, *PPM1F*, *PCDP1*, *ATP2A1*, *GPR171*, and *RP1-34H18.1-1*) were consistent with RNA sequencing results. We suggest that, from among these eleven genes, two upregulated genes, *CPNE3* and *OPHN1*, and one downregulated gene, *PPM1F*, play an important role in PDL regeneration in humans when orthodontic force is applied.

## 1. Introduction

Periodontal ligament (PDL), which is a group of connective tissue fibers, connects the tooth root to the adjacent alveolar bone. It protects blood vessels and nerves by absorbing mechanical force such as mastication force and provides proprioception. Moreover, it mediates orthodontic tooth movement under compressional or tensional force and plays a critical role in recovery from periodontal disease [1]. Mesenchymal stem cells were isolated from PDL [2] and have shown potential for periodontal regeneration [3]. PDL regeneration can also be enhanced under mechanical stimulus [4,5,6]. Low-magnitude, high frequency mechanical vibration promotes human PDL stem cells differentiation [7], and mechanical shear stress promotes the osteogenic differentiation of dental stem cells, including those derived from the pulp and the PDL [8]. An in vivo study using rats showed that occlusal stimulus promotes regeneration of PDL and prevents dentoalveolar ankylosis after autotransplantation [9].

Changes in PDL cells under mechanical stress have been reported to be critical in maintaining homeostasis of the periodontal tissue and enabling its remodeling [10,11,12,13]. Cyclic stretch applied to the PDL influences membrane protein expression [10] and upregulates and downregulates genes related to the extracellular matrix in cultured human PDL cells [11]. Orthodontic force activates the CC chemokine receptor 5 (CCR5)-CCR5 ligands axis in rats, which was suggested to be a key factor in PDL remodeling. Moreover, PDL induces favorable circumstances for remodeling by releasing cytokines and growth factors under orthodontic force in animal models [12,13]. However, there have been few in vivo studies investigating the relation between mechanical stimulus and PDL regeneration in humans. Although PDL exhibits different length, volume, mechanoreceptors, and remodeling capacity according to occlusal contact [14], it would be meaningful to investigate the transcriptional changes in PDL under mechanical stimulus.

Early application of the mechanical force can increase the success rate of autotransplantation by preventing ankylosis and external root resorption, which is based on PDL regeneration [15]. Under mechanical stress, expression of cell cycle regulatory proteins, such as proliferating cell nuclear antigen, cyclin-dependent kinases, and cyclin D1, are increased in human PDL cells [16], although transcriptional changes are barely unknown in humans. RNA sequencing (RNA-seq) is a useful technique for analyzing dynamic transcriptomes rather than static genomes [17] and discovering novel transcripts from a wide range of transcriptomes as well as providing quantitative analysis of gene expression [18].

In the present study, we simulated mechanical stimulus to the PDL by applying orthodontic force. If mechanical stimulus induces changes in PDL gene expression in humans, RNA-seq can show these changes in transcriptional expression patterns, which would prove valuable in understanding the changes occurring in PDL following mechanical stimulus such as mastication force, orthodontic force, or trauma to the teeth, and determining potential target genes. Therefore, the aims of this study were to investigate changes in gene expression in the PDL after mechanical stimulus and to search for novel genes through RNA sequencing.

## 2. Materials and Methods

### 2.1. Patient Selection

Subjects free from underlying disease were selected from orthodontic patients who had visited Yonsei University Dental Hospital between June 2017 and May 2018 for orthodontic extraction of at least two premolars. The inclusion criteria were as follows: a sound premolar in one quadrant without caries or restorations, and no history of taking any steroidal or non-steroidal anti-inflammatory drugs during force application. The exclusion criteria were root malformation such as dilaceration, which might result in difficult extraction. The minimum sample size of 10 subjects was estimated to overcome the individual variation in gene expression in PDL cells on the basis of a previous study using RNA-seq to analyze differences in gene expression between periodontitis-affected and healthy sides [19]. The study protocol was approved by the institutional review board of Yonsei University Dental Hospital (IRB No. 2-2017-0028) and performed after obtaining written informed consent from every participant.

Based on the inclusion/exclusion criteria, 14 patients were initially enrolled. Samples from three patients were excluded at initial quality check, and the samples from 11 subjects (5 men and 6 women; mean age, 22.4 years; age range, 17.5–31.0 years) were used in this study (Table A1). Two premolars were extracted in seven subjects; three premolars (two premolars in the experimental group and one premolar in the control group) were extracted in one subject (subject #4); and four premolars (two premolars in the experimental group and two premolars in the control group) were extracted in three subjects (subjects #7, #8, and #9). In cases of 3- or 4-premolar extraction, the two teeth on the same side were assigned to the same group.

### 2.2. Mechanical Stimulus (Applying Orthodontic Force)

**Figure 1:**
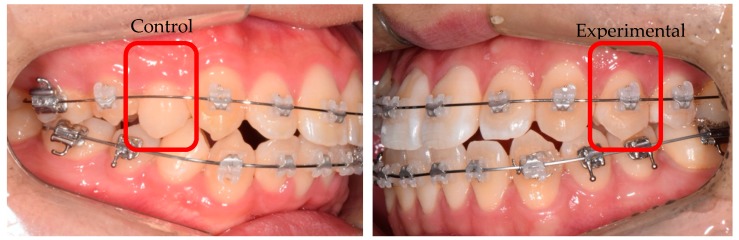
Split-mouth design: control group (left, no orthodontic force) and experimental group (right, orthodontic force applied).

To stimulate the PDL, orthodontic force was applied to the first or second premolar on one side (the experimental group; *n* = 11), whereas the tooth on the other side was left untreated (the control group; *n* = 11). The study was a split-mouth, randomized, controlled trial with a 1:1 allocation ratio. A 0.016-inch nickel–titanium wire (Tomy International, Yokohama, Japan), which is known to deliver light continuous force (100*× g*) with a deflection of 0.5–1.8 mm, was used to apply mechanical stimulus in the experimental group (Figure 1). The orthodontic force was applied for three weeks [20], and thereafter, the teeth in both groups were carefully extracted. PDL cells were washed with cold PBS after gentle scrapping from the middle third of the root and prepared for RNA-seq.

### 2.3. RNA Sequencing

To evaluate changes in gene expression after mechanical stimulus, total RNA was extracted from PDL cells using TRIzol Reagent (Invitrogen, Waltham, MA, USA). The RNA was stored at −70 °C and measured at an optical density of 260 nm. The mixtures of total RNA were incubated with Oligo dT (Gibco BRL, Rockville, NY, USA).

The library was constructed, sequenced using an Illumina HiSeq2500 sequencer (Illumina, CA, USA), and the data obtained through RNA-seq. A gene set underlying mechanical stimulus was analyzed using gene set enrichment analysis (GSEA). Differentially expressed gene (DEG) analysis was also performed to determine differences in gene expression between the experimental and control groups. For GSEA, libraries from 11 subjects in each experimental or control group were merged into one library for each group, and then, the experimental and control groups in total, were compared. Based on the GSEA results, an enrichment map was obtained. DEG analyses were performed to compare the libraries between the experimental and control groups in each sample as well as in the merged samples. Based on the DEG results, we selected target genes based on gene expression fold-changes: more than 1.5 fold-change in gene expression in the experimental group compared to the control group was defined as upregulated, and less than −1.5 fold-change was defined as downregulated [21,22,23].

### 2.4. Real-Time PCR

Real-time PCR was performed for the selected upregulated and downregulated genes to verify RNA-seq results. A cDNA synthesis reaction was performed using a mixture of AccuPower PCR PreMix (Bioneer, Daejeon, Korea) and water. The cDNA was synthesized from total RNA obtained from both groups using the SuperScript First-Strand Synthesis System (Invitrogen, Carlsbad, CA, USA) and was amplified with an ABI-7300 (Applied Biosystems, Mortlake, Waltham, Massachusetts, USA). The amplified cDNA was detected with SYBR Green PCR Master Mix Reagent Kit (Takara, Seoul, Korea). PCR conditions were as follows: incubation for 10 min at 95 °C, followed by 40 cycles of 10 s denaturation at 95 °C, and annealing for 60 s at 60 °C. The reaction mixture lacking cDNA was used as a negative control in each run. Primer sequences are summarized in Table 1. Ratios of the intensities of the target genes and GAPDH signals were used as a relative measure of the expression level of the target genes. To ensure accuracy of the experiments, primer specificity was confirmed by the dissociation curve after PCR, and real-time PCR assays were performed in triplicate for each sample. The mean fold-change in expression in the experimental group compared with the control group was calculated from the △△Ct values, and the range of the fold-changes was represented by standard deviations of the values [21].

### 2.5. Statistical Analysis

For differential expression analysis, gene level count data were generated using HTSeq-count v0.5.4p3 tool [22] with the option “-m intersection-nonempty” and -r option considering paired-end sequence. Based on the calculated read count data, DEGs were identified using the R package called TCC [23]. TCC package applies robust normalization strategies to compare tag count data. Normalization factors were calculated using the iterative DEGES/edgeR method. Q-value was calculated based on the p-value using the p.adjust function of R package with default parameter settings. Differentially expressed genes were identified based on the q value threshold less than 0.05.

## 3. Results

### 3.1. Extracted Premolar Samples

Two premolars were extracted in seven subjects; three premolars (two premolars in the experimental group and one premolar in the control group) were extracted in one subject (subject #4); and four premolars (two premolars in the experimental group and two premolars in the control group) were extracted in three subjects (subjects #7, #8, and #9). In cases of 3- or 4-premolar extraction, the two teeth on the same side were assigned to the same group.

### 3.2. Gene Set Enrichment Analysis of Mechanically Stimulated PDL Cells

**Figure 2:**
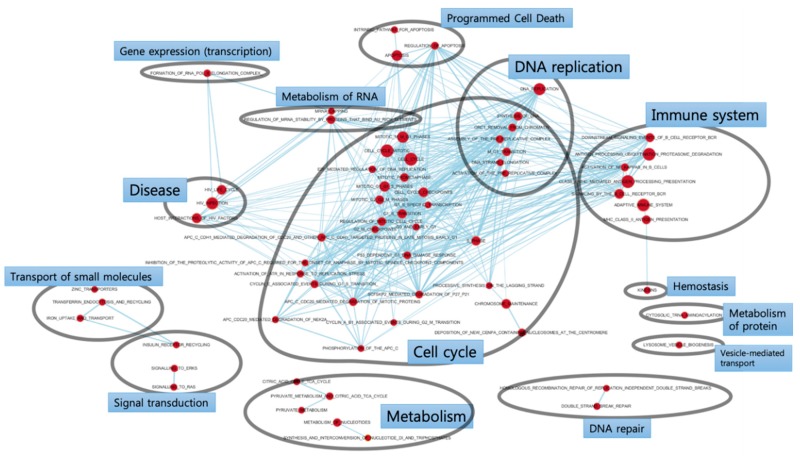
Gene set enrichment analysis (GSEA) enrichment map. The enriched gene sets in the periodontal ligament underlying mechanical stimulus are mostly related to the cell cycle pathway.

GSEA enrichment maps show enriched gene sets, most of which are related to the cell cycle pathway (Figure 2). Additionally, pathways for DNA replication, immune system, and metabolism were represented among upregulated genes. The node size, which indicates the number of enriched genes, was large in the following gene sets: cell cycle, cell cycle mitotic, DNA replication, antigen processing ubiquitination proteasome degradation, and class I MHC-mediated antigen processing presentation.

### 3.3. DEG Analysis of Mechanically Stimulated PDL Cells

**Figure 3:**
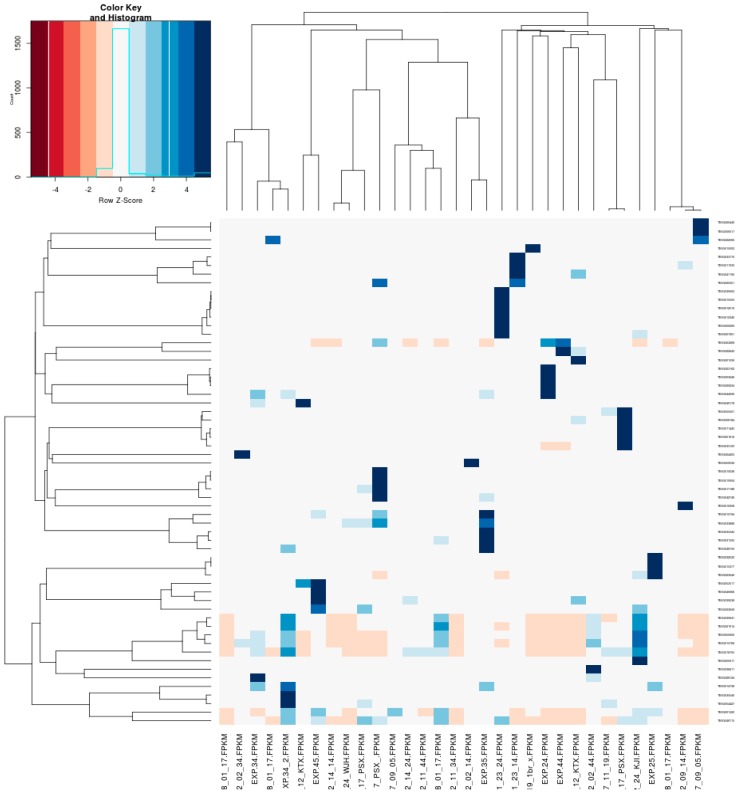
Heatmap representing differential expression of the periodontal ligament underlying mechanical stimulus. This heatmap demonstrates the Fragments Per Kilobase Million (FPKM) value of the most significant differentially expressed genes. FPKM value indicates the relative expression of a transcript which is proportional to the number of cDNA fragments in RNA sequencing. A detailed heatmap for each subject is shown in the appendix.

**Figure A1:**
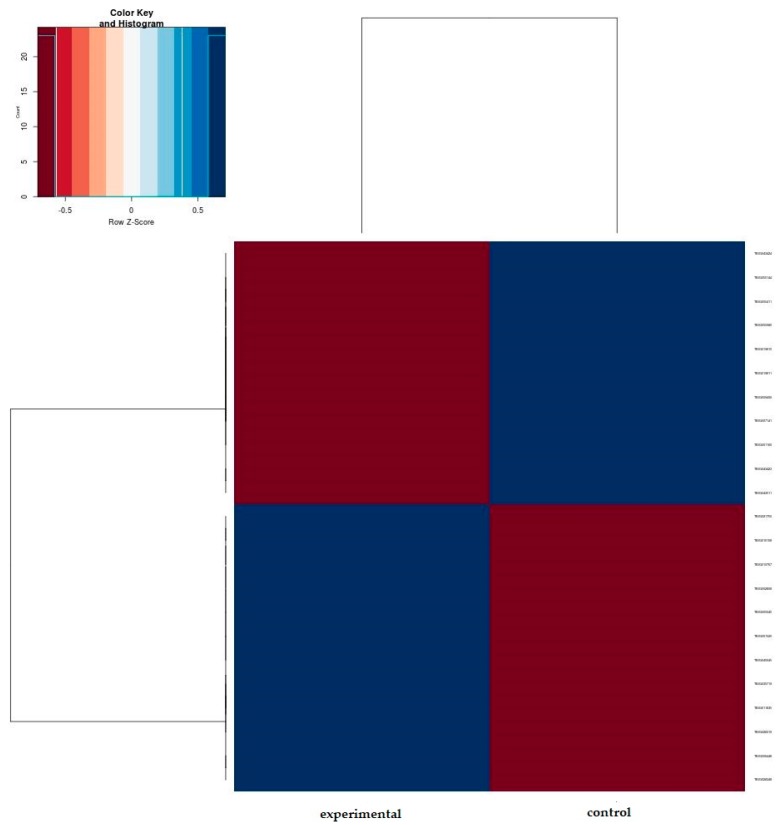
Heatmap of subject #1. Experimental group vs. control group.

**Figure A2:**
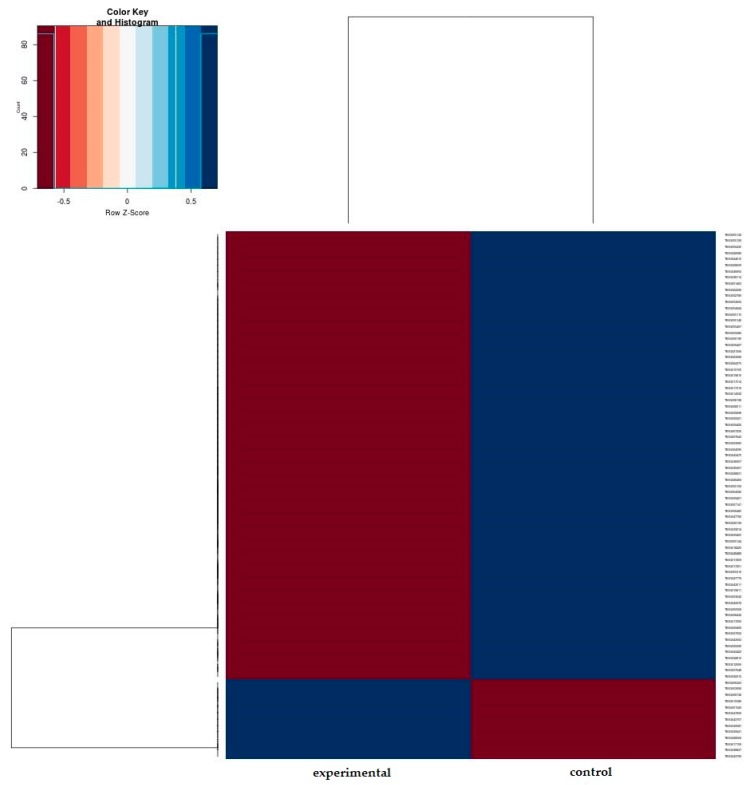
Heatmap of subject #2. Experimental group vs. control group.

**Figure A3:**
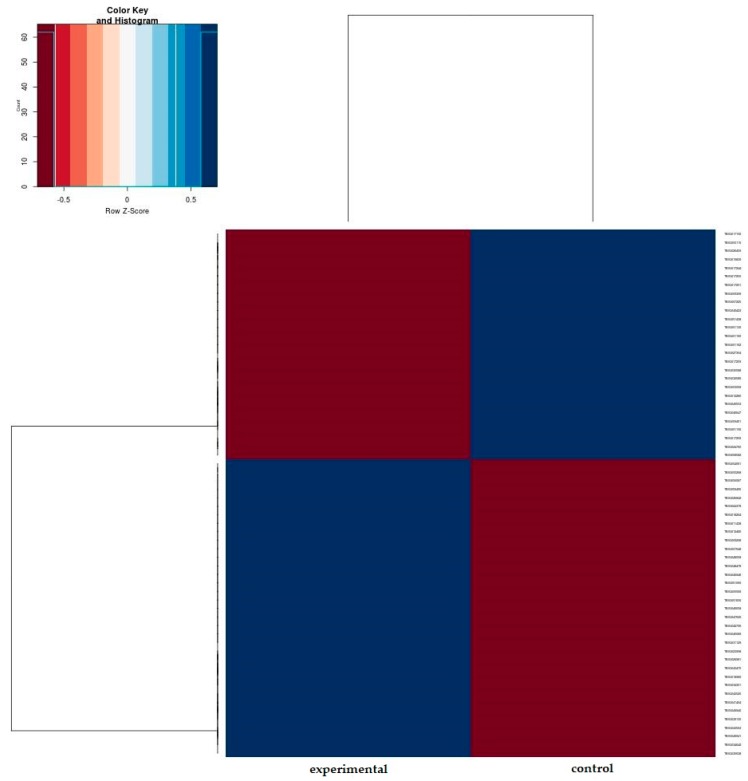
Heatmap of subject #3. Experimental group vs. control group.

**Figure A4:**
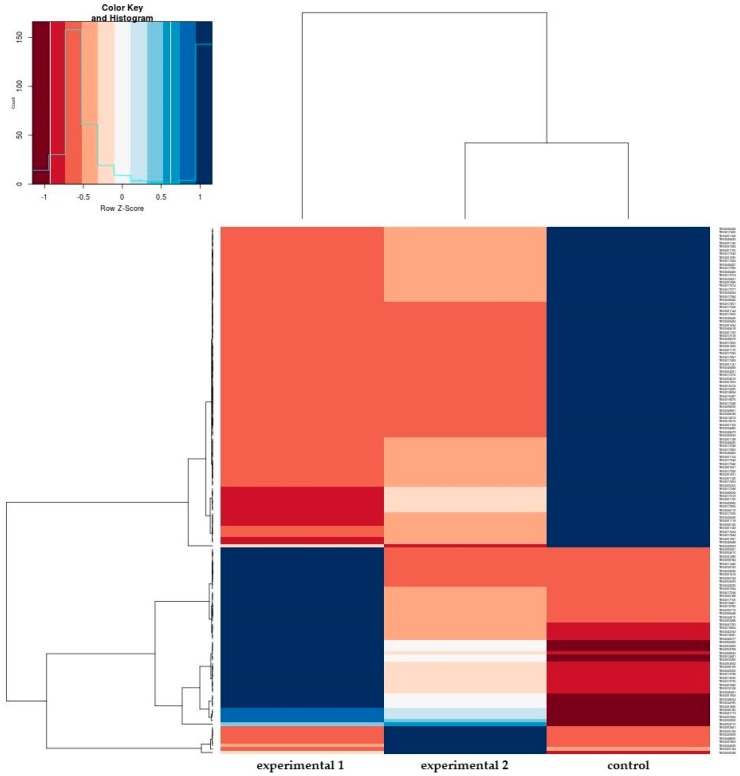
Heatmap of subject #4. Experimental group vs. control group.

**Figure A5:**
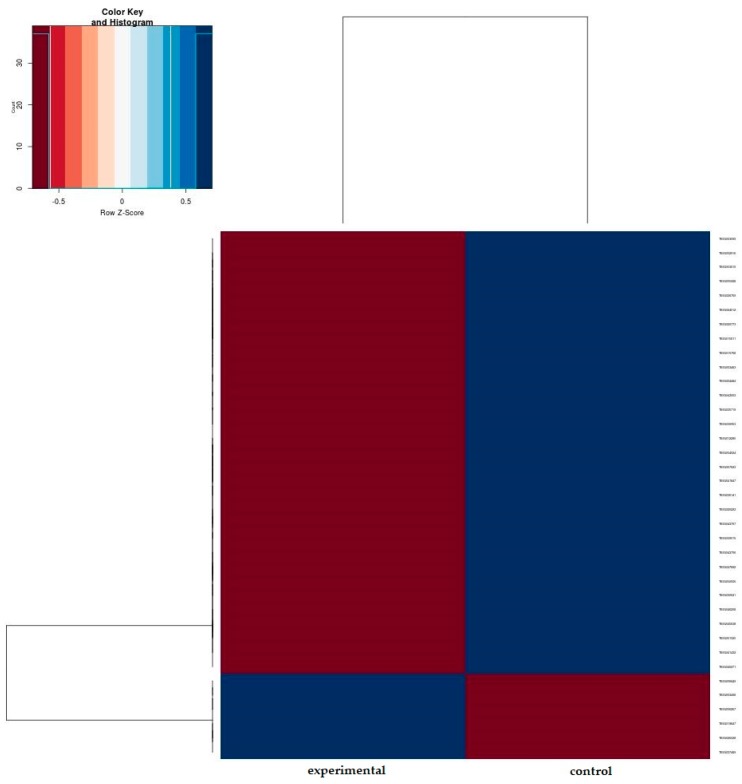
Heatmap of subject #5. Experimental group vs. control group.

**Figure A6:**
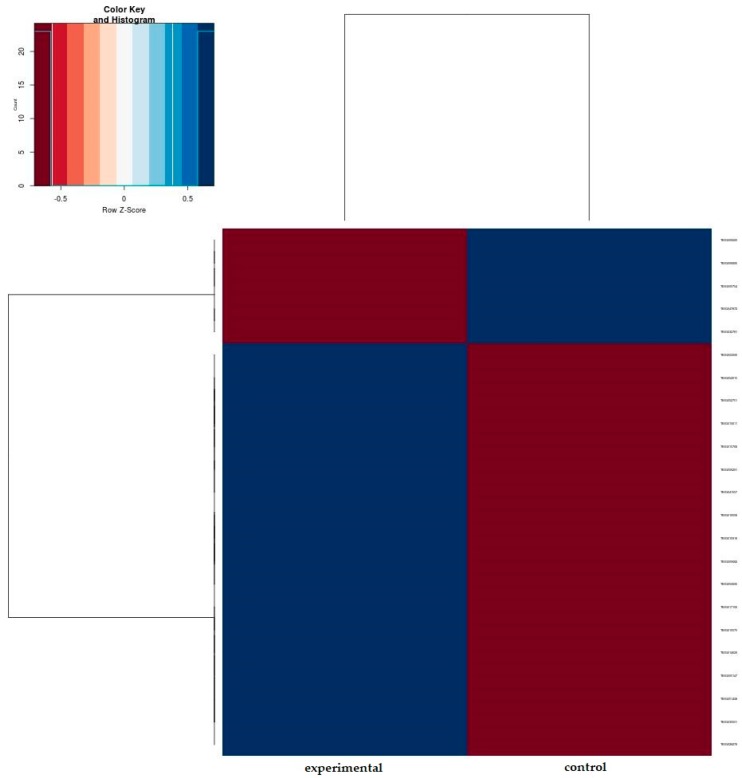
Heatmap of subject #6. Experimental group vs. control group.

**Figure A7:**
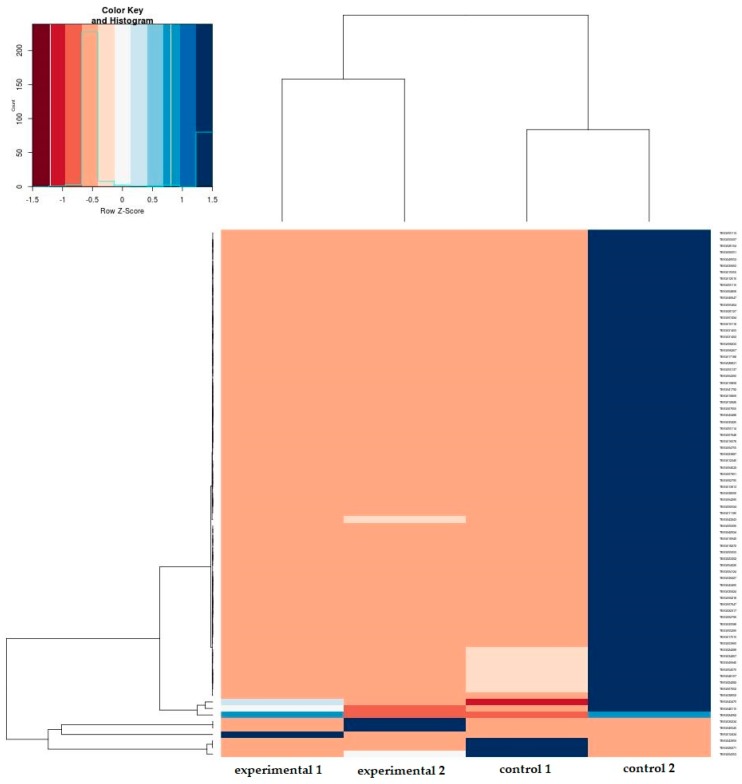
Heatmap of subject #7. Experimental group vs. control group.

**Figure A8:**
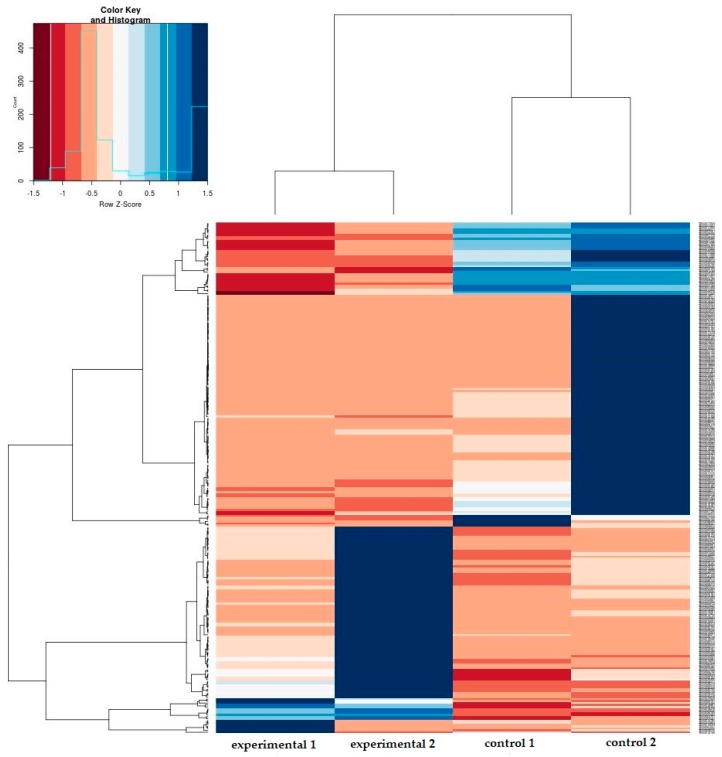
Heatmap of subject #8. Experimental group vs. control group.

**Figure A9:**
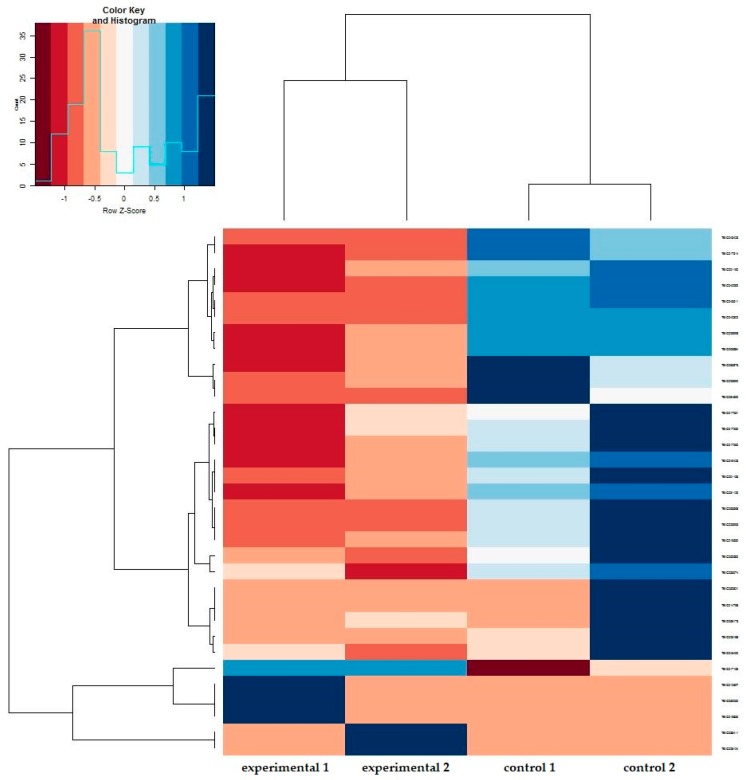
Heatmap of subject #9. Experimental group vs. control group.

**Figure A10:**
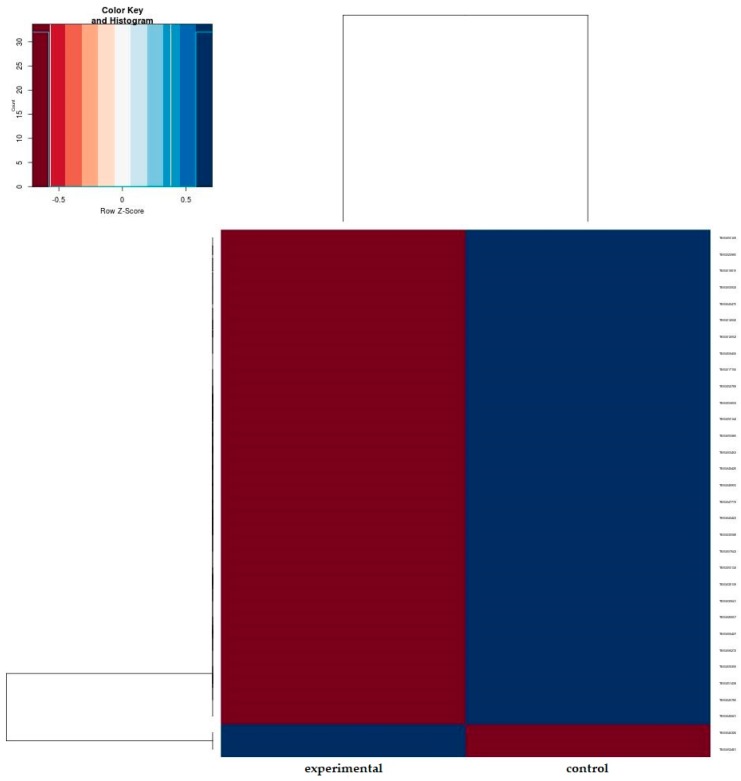
Heatmap of subject #10. Experimental group vs. control group.

**Figure A11:**
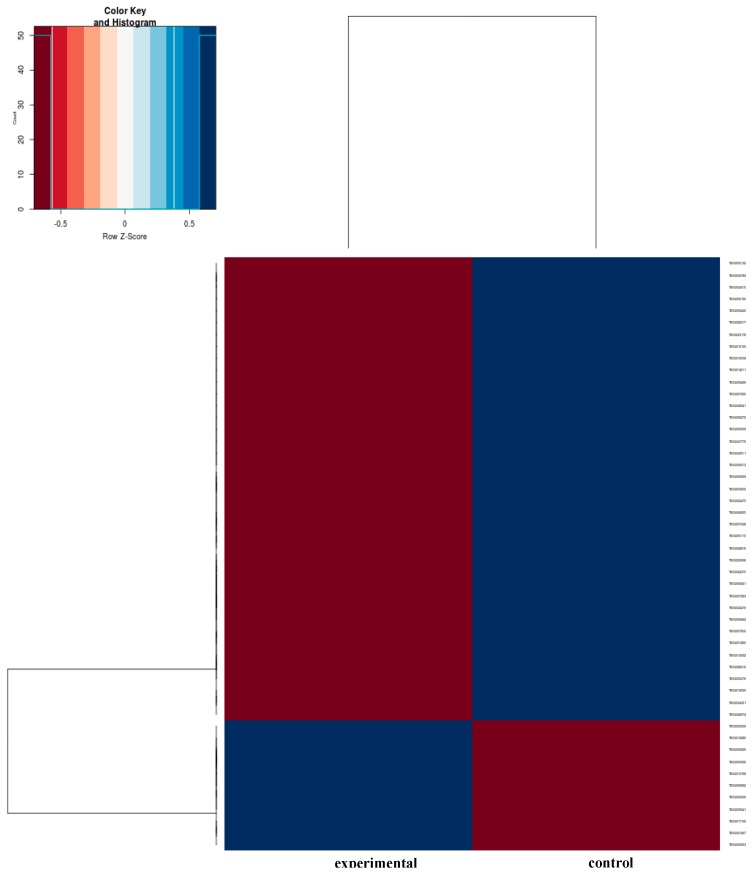
Heatmap of subject #11. Experimental group vs. control group.

A heatmap was obtained by comparing the merged libraries of 11 subjects between the experimental and control groups (Figure 3). The 11 heatmaps obtained from each subject are presented in the Appendix Materials (Figure A1, Figure A2, Figure A3, Figure A4, Figure A5, Figure A6, Figure A7, Figure A8, Figure A9, Figure A10 and Figure A11). Fifty-nine genes were selected by *q* value (*q* < 0.05), then 13 up-regulated and 20 down-regulated genes were finally selected based on a fold-change of 1.5 (Table 2 and Table 3).

### 3.4. Validation of DEGs in Mechanically Stimulated PDL Cells

**Figure 4:**
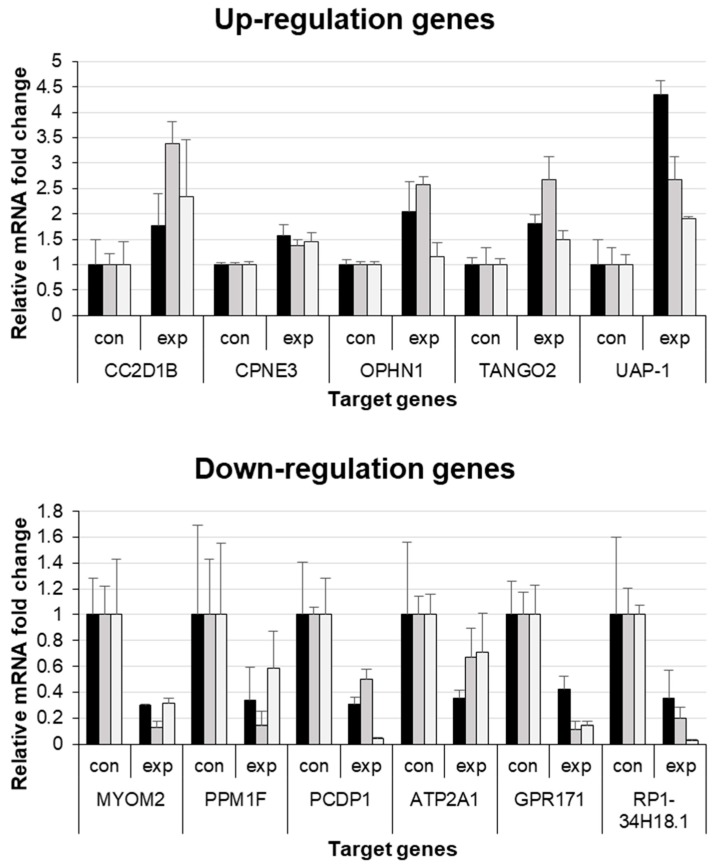
Real-time quantitative PCR results of the 5 upregulated and 6 downregulated genes from RNA sequencing. Con, control group; exp, experimental group.

Among the 13 upregulated and 20 downregulated genes, we performed gene searches in the human gene database (www.genecard.org) and selected 5 upregulated (*CC2D1B*, *CPNE3*, *OPHN1*, *TANGO2*, and *UAP-1*) and 6 downregulated (*MYOM2*, *PPM1F*, *PCDP1*, *ATP2A1*, *GPR171*, and *RP1-34H18.1-1*) genes related to the cell cycle. Real-time PCR results for the 5 upregulated and 6 downregulated genes confirmed the corresponding RNA-seq results (Figure 4).

## 4. Discussion

This prospective study investigated the transcriptional expression in human PDL cells stimulated with an orthodontic force with RNA-seq. Because we intended to identify gene transcripts potentially related to the remodeling of PDL, this study focused on the differential expression of PDL genes using GSEA, DEG analysis, and real-time PCR. From these results, we found 11 significant DEGs when orthodontic force was applied on the PDL.

In the early phase of orthodontic tooth movement, mechanically stimulated PDL cells recruit local factors related to homeostasis and remodeling [16,24] of the surrounding periodontal tissue, which may result from the upregulated cell cycle pathways. Under tension force, PDL cells participate in DNA synthesis of osteoblasts; are differentiated to osteoblasts; or are released from G_2_ block, which contributes to the alveolar bone formation. Moreover, morphological deformation of the PDL induced by mechanical force plays a key role in recovery of its original shape via the remodeling process [25]. Therefore, the upregulated cell cycle of the PDLs would make orthodontic tooth movement possible by facilitating the remodeling process.

Among the 11 genes, *CPNE3*, *OPHN1*, and *PPM1F* are specially interesting, which were likely related to PDL remodeling based on the function and the signaling pathway of each gene. CPNE3 is a calcium-dependent membrane-binding protein that plays a major role in the synthesis of phospholipids and the immune system. CPNE3 belongs to the copine family, which can bind both calcium ions and phospholipids simultaneously. In particular, *CPNE7* is involved in PDL regeneration [26]. The gene has a functional role in attachment of PDL cells to cementum and promotes the physiological arrangement of PDL fibers. Therefore, *CPNE3*, in the same gene family as *CPNE7*, may be involved in the regeneration of PDL. Further studies are needed to investigate whether and how CPNE3 affects PDL regeneration.

*OPHN1* encodes the protein oligophrenin-1 that has a Rho-GAP domain shown to negatively regulate RhoA, Rac, and Cdc42 in vitro and in nonneuronal cells [27]. Rho GTPases participate in important cell biological processes, including cell growth control, cell motility, and development [28]. As expression of *OPHN1* increases, the rhoA, Rac, and Cdc42 proteins, which are known to activate caspase 3 and finally activate apoptosis, decrease, which leads to reduced apoptosis of PDL cells. Therefore, upregulation of *OPHN1* may indirectly contribute to the maintenance of PDL cells.

*PPM1F* is a member of the serine/threonine protein phosphatase family. This gene is known to be involved in caspase-dependent apoptosis [29]. When orthodontic force is applied, apoptosis in PDL cells may be inhibited by downregulation of *PPM1F*. It can be assumed that downregulation of *PPM1F* has a positive effect on PDL regeneration.

The upregulated and downregulated genes identified in the DEG analysis were confirmed by qPCR, although the qPCR ratio of each sample was different. Furthermore, there was a great variety of gene expression between subjects. It was assumed that limitations of the clinical study using human samples might cause the variety. As seen in the demographic features of the subjects (Table A1), different age, sex, tooth allocation, and occlusion of each subject would result in a wide variety of gene expressions unlikely in the animal models. In addition, the orthodontic force in the experimental group and the occlusal force in the control group would not be exactly the same in each tooth, which may result in differences in gene expression [4]. Moreover, the nonlinear properties of the PDL under different loading directions [30] would affect the outcome. The unstable nature of RNA and different elapsed times from tooth extraction to RNA extraction from the PDL may be another reason for the difference because proliferation of PDL fibers changes depending on post-extraction time [31].

The small numbers of DEG, 13 upregulated and 20 downregulated genes, were selected by comparing the merged libraries of 11 subjects between the experimental and control groups in the present study. Although the GSEA analysis demonstrated not only the cell cycle pathways but also the pathways related to metabolism, immune system, disease, and programmed cell death, the present study focused only on the genes related to the cell cycle. There are other factors that limited the number of DEG: high cutoff value, short duration of mechanical stimulation, and non-differentiation between compression and tension sides of the root surface, which might prevent the genes involved in PDL regeneration from expressing differentially. Moreover, the 33 genes could not be fully verified by qPCR in this study. Although 22 genes were discarded because they were not related to the cell cycle, some of the genes might play a role in PDL regeneration indirectly.

Mechanical stimulus contributes to PDL regeneration in vitro [4,7] and prevention of ankylosis after transplantation or replantation in in vivo animal models [9]. To the best of our knowledge, this is the first report of gene expression changes in the PDL following mechanical stimulus, in humans. We confirmed the upregulation of the cell cycle pathways after mechanical stimulus and found novel genes related to the process. Future studies are needed to identify the biological role of these genes and to obtain clinical relevancies such as saving the tooth using mechanical stimulus in trauma or transplantation cases.

## 5. Conclusions

When orthodontic force is applied, gene sets related to the cell cycle pathway were upregulated in PDL. The 5 upregulated (*CC2D1B*, *CPNE3*, *OPHN1*, *TANGO2*, and *UAP-1*) and 6 downregulated genes (*MYOM2*, *PPM1F*, *PCDP1*, *ATP2A1*, *GPR171*, and *RP1-34H18.1-1*) genes may play an important role in PDL regeneration after mechanical stimulus in humans.

## Figures and Tables

**Table 1:** Primer sequences used for real-time quantitative PCR.

Genes	Transcript ID	Forward	Reverse
CC2D1B	NM_032449.2	GAGTCGCAGCTAGCCTCTGT	TCTGTCTCAGGGCTCCTGTT
TMEM253	NM_001146683.1	CTTGCTGAGCCAGAGGAAAC	CAAACCAGGAACCTCTTCCA
TENM4	NM_001098816.2	CCGTCTTCCTTTCTGACAGC	ATCAGCCCAAACTTGTCCAC
CPNE3	NM_003909.5	TCGACCACTGGTGATGAAAA	CCGATGAACCATTAGCCAGT
MYOM2	NM_003970.4	CGGTGAATACAAGGCAACCT	TCACATATCTGCAGCCAAGC
PPM1F	NM_014634.4	GTACAGCAGGGACAGGTGGT	ACAGGCAAGCAGCAGGTAGT
PCDP1	NM_001271049.2	TCAACAAGTAGCACGCAAGG	ATCCGCCTCAGGAAGAATTT
ATP2A1	NM_173201.3	TGGCTCTTCTTCCGCTACAT	GCCTCGAAGACCTCACAGTC
GPR171	NM_013308.3	CAACCGTTGTGTGGCTAATG	TATGATGTAGCCCGTGGTCA
OPHN1	NM_002547.3	GTCCCCAAGCAGGCCTAT	GTCCATTGGTGGCCTTTG
TANGO2	NM_152906.6	TCCCTGGAGGAAGCTGTG	GCTGCGCCTCTTCATTGT
UAP1	NM_001324116.1	TCCAAAGCTGGGCAAGAG	GGTTCCATTCGTGCATCC
RP1-34H18.1	ENST00000550042.1	GCGGAGGAGGGAAGAAAG	AAAACCAACCGAGGCACA
GAPDH		TCCGCGGCTATATGAAAACAG	TCGTAGTGGGCTTGCTG AA

**Table 2:** RNA sequencing results: 13 upregulated genes after mechanical stimulus in the periodontal ligament (fold-change ≥ 1.5).

Gene Name	Description	Control	Experimental	log2fc	*p* Value	*q* Value
**OPHN1**	oligophrenin1	5.31	781	7.2	5.00E-05	0.0149
**TANGO2**	transport and golgi organization 2 homolog (Drosophila)	6.66	86.1	3.69	5.00E-05	0.0149
**CC2D1B**	coiled-coil and C2 domaincontaining 1B	12	150	3.58	5.00E-05	0.0149
**UAP1**	UDP-N-acteylglucosaminepyrophosphorylase1	43	468	3.45	5.00E-05	0.0149
**TMEM253**	transmembrane protein 253	0.836	7.66	3.2	5.00E-05	0.0149
**TENM4**	teneurin transmembrane protein 4	13.5	93.6	2.8	5.00E-05	0.014911
**ABHD4**	abhydrolase domain containing 4	20.9	137	2.71	5.00E-05	0.0149
**CPNE3**	copine III	23.7	134	2.5	5.00E-05	0.0149
**RP11-820L6.1**	-	1.76	9.19	2.38	5.00E-05	0.0149
**SPATA22**	spermatogenesis associated 22	0.379	1.89	2.31	5.00E-05	0.0149
**SCUBE1**	signalpeptide, CUB domain, EGF-like1	0.217	0.88	2.02	0.0001	0.0277
**ARMC8**	armadillo repeat containing 8	13.5	54	2	5.00E-05	0.0149
**ERI2**	ERI1 exoribonuclease family member 2	6.69	26.5	1.99	5.00E-05	0.0149

**Table 3:** RNA sequencing results: 20 downregulated genes after mechanical stimulus in the periodontal ligament (fold-change ≤ −1.5).

Gene Name	Description	Control	Experimental	log2fc	*p* Value	*q* Value
**ITCH**	itchy E3 ubiquitin protein ligase	41.6	14.4	−1.53	5.00E-05	0.0149
**ARL14EP**	ADP-ribosylation factor-like 14 effector protein	71.8	22.8	−1.65	5.00E-05	0.0149
**RFX1**	regulatory factor X, 1 (influences HLA class II expression)	17.1	5.47	−1.65	5.00E-05	0.0149
**PLEKHH1**	pleckstrin homology domain containing, family H (with MyTH4 domain) member1	6.91	2.18	−1.66	5.00E-05	0.0149
**EHBP1L1**	EH domain binding protein1-like 1	162	46	−1.81	5.00E-05	0.0149
**PIGQ**	phosphatidylinositol glycan anchor biosynthesis, class Q	286	81.7	−1.81	5.00E-05	0.0149
**ASPG**	asparaginase homolog (S.cerevisiae)	56.8	15.2	−1.9	5.00E-05	0.0149
**BOD1L1**	biorientation of chromosomes in cell division 1-like 1	92.5	22.5	−2.04	5.00E-05	0.0149
**PTPN12**	protein tyrosine phosphatase, non-receptortype12	268	55.9	−2.26	5.00E-05	0.0149
**USH1C**	Usher syndrome 1C(autosomal recessive,severe)	4.9	0.927	−2.4	5.00E-05	0.0149
**MYOM2**	myomesin 2	18.2	3.03	−2.58	5.00E-05	0.0149
**PRR11**	proline rich11	16.7	2.72	−2.62	5.00E-05	0.0149
**CCDC91**	coiled-coil domain containing 91	166	26.8	−2.63	5.00E-05	0.0149
**PPM1F**	protein phosphatase,Mg2+/Mn2+ dependent, 1F	165	24.5	−2.75	5.00E-05	0.0149
**PCDP1**	Homo sapiens primary ciliary dyskinesia protein1 (PCDP1),transcript variant1, mRNA.	2.35	0.254	−3.21	5.00E-05	0.0149
**TSPAN8**	tetraspanin 8	34.8	3.1	−3.49	5.00E-05	0.0149
**RCCD1**	RCC1 domain containing1	114	7.76	−3.87	5.00E-05	0.0149
**ATP2A1**	ATPase, Ca++ transporting,cardiac muscle,fast twitch 1	13.7	0.916	−3.91	5.00E-05	0.0149
**GPR171**	G protein-coupled receptor 171	49	0.782	−5.97	5.00E-05	0.0149
**RP1-34H18.1**	-	4.14E+03	0.774	−12.4	5.00E-05	0.0149

## Appendix A

**Table A1:** Demographic features of subjects.

Subject No.	Sex	Age	Tooth allocation	
			Experimental Group	Control Group
1	Female	28 years 2 months	Mx Lt 2nd premolar	Mn Lt 2nd premolar
2	Male	22 years 0 months	Mx Lt 1st premolar	Mn Lt 1st premolar
3	Male	17 years 6 months	Mx Rt 1st premolar	Mx Lt 1st premolar
4	Male	20 years 3 months	Mx Lt 2nd premolar, Mn Lt 2nd premolar	Mn Rt 1st premolar
5	Female	20 years 9 months	Mn Lt 1st premolar	Mx Lt 1st premolar
6	Female	25 years 7 months	Mn Lt 1st premolar	Mx Lt 1st premolar
7	Female	31 years 0 months	Mx Lt 1st premolar, Mn Lt 1st premolar	Mx Rt 1st premolar, Mn Rt 1st premolar
8	Female	20 years 8 months	Mx Rt 1st premolar, Mn Rt 1st premolar	Mx Lt 1st premolar, Mn Lt 1st premolar
9	Male	18 years 4 months	Mx Lt 1st premolar, Mn Lt 1st premolar	Mx Rt 1st premolar, Mn Rt 1st premolar
10	Female	18 years 6 months	Mx Lt 1st premolar	Mx Rt 1st premolar
11	Male	25 years 1 months	Mx Rt 1st premolar	Mn Rt 1st premolar

Mx, maxilla; Mn, mandible; Rt, right side; Lt, left side.
